# Ventricular Tachycardia Ablation in Structural Heart Disease With LVEF > 35%: Clinical Outcomes and Recurrence Patterns

**DOI:** 10.1111/jce.70455

**Published:** 2026-07-19

**Authors:** Said‐Elias Waezsada, Moneeb Khalaph, Martin Braun, Thomas Fink, Vanessa Sciacca, Nadica Trajkovska, Philipp Lucas, Ersan Akkaya, Maxim Didenko, Mustapha El Hamriti, Guram Imnadze, Denise Guckel, Christian Sohns, Philipp Sommer, Angeliki Darma

**Affiliations:** ^1^ Department of Electrophysiology, Heart and Diabetes Center NRW Ruhr University Bochum Bad Oeynhausen Germany; ^2^ Clinic for Cardiology/Electrophysiology, GZO‐Spital Wetzikon Zürich Switzerland; ^3^ Clinic for Electrophysiology, University Hospital Ruppin‐Brandenburg Germany

**Keywords:** catheter ablation, moderate ejection fraction, structural heart disease, ventricular tachycardia

## Abstract

**Background:**

In patients with structural heart disease (SHD) and moderately impaired left ventricular ejection fraction (LVEF > 35%), data on outcomes after ventricular tachycardia (VT) ablation remain limited. This analysis focuses on VT recurrence after ablation in patients presenting with sustained VT and LVEF > 35% within a secondary‐prevention population.

**Objective:**

To evaluate procedural outcomes and long‐term VT recurrence after catheter ablation in SHD patients with LVEF > 35%.

**Methods:**

We analyzed 219 consecutive patients with SHD and LVEF > 35% undergoing VT ablation, including 89 with ischemic cardiomyopathy (ICM) and 130 with non‐ischemic cardiomyopathy (NICM). Procedural characteristics, complications, and VT recurrence during follow‐up were compared between groups.

**Results:**

ICM patients were older, more frequently hypertensive, and had slightly lower LVEF than NICM patients. Ablation was predominantly endocardial in ICM, whereas combined endocardial–epicardial ablation was required in 28% of NICM patients (*p* < 0.001). Acute VT non‐inducibility was achieved more frequently in ICM than in NICM (93% vs 67%; *p* < 0.001). Procedural complications were infrequent and comparable between groups (6% overall; *p* = 0.342). During follow‐up, VT recurred in 35% of patients, more frequently in NICM than ICM (42% vs 25%; *p* = 0.004). Cardiomyopathy type emerged as the only independent predictor of VT recurrence (HR 2.312, CI 1.3–4.0, *p* = 0.004), while acute non‐inducibility was associated with a lower recurrence risk.

**Conclusion:**

VT ablation in SHD patients with LVEF > 35% was associated with acceptable safety and moderate arrhythmia control. Outcomes were more favorable in ICM than in NICM, reflecting the heterogeneity of arrhythmic risk in this secondary prevention population.

## Graphical abstract

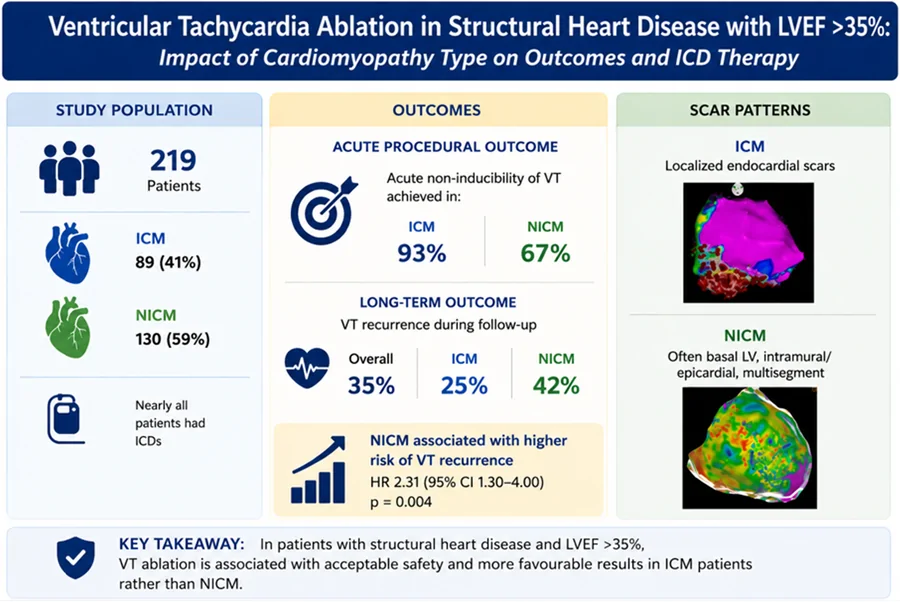

**Abbreviations**
ATP: anti‐tachycardia pacing
BMI: body mass index
ECMO: extracorporeal membrane oxygenation
HF: heart failure
HTX: heart transplantation
ICD: implantable cardioverter‐defibrillator
ICM: ischemic cardiomyopathy
LBB: left bundle brunch
LVEF: left ventricular ejection fraction
NICM: non‐ischemic cardiomyopathy
SCD: sudden cardiac death
SD: standard deviation
SHD: structural heart disease
VT: ventricular tachycardia
(L/R)VAD: (left/right) ventricular assist device

## 1 Introduction

Patients with structural heart disease (SHD), whether of ischemic or non‐ischemic origin, remain at substantial risk for sustained ventricular tachycardia (VT) and sudden cardiac death (SCD) despite advances in medical therapy and catheter ablation techniques [1]. Current guidelines recommend implantable cardioverter‐defibrillator (ICD) therapy for secondary prevention in patients presenting with sustained VT, irrespective of left ventricular ejection fraction (LVEF) [2, 3]. While this recommendation is well established, real‐world data on arrhythmic outcomes after catheter ablation in patients with SHD and LVEF > 35% remain limited [4]. In particular, the extent to which VT recurrence patterns and procedural outcomes differ according to cardiomyopathy etiology in this population has not been well characterized.

Although preserved LVEF has traditionally been associated with a lower risk of arrhythmic events, recent data indicate that patients with SHD and LVEF > 35% may continue to experience significant rates of VT recurrence after ablation [5]. In contemporary series and pooled analyses, recurrence rates ranging from 20% to 30% have been reported within 2 years, even among patients achieving acute procedural success [6]. These findings suggest that LVEF alone may be insufficient for post‐ablation risk stratification and that additional substrate‐ or procedure‐related parameters may identify individuals who remain vulnerable to malignant arrhythmias [7].

The present study aims to describe procedural outcomes and VT recurrence after catheter ablation in SHD patients with LVEF > 35%, with a focus on differences between ischemic and non‐ischemic cardiomyopathy.

## 2 Methods

### 2.1 Study Design and Population

This observational cohort study included 219 consecutive SHD patients who underwent catheter ablation for sustained VT between 2019 and 2025 at our tertiary, high‐volume electrophysiology referral center. Eligible patients had a documented sustained ventricular tachycardia according to current guideline definitions, including VT episodes lasting ≥ 30 s as well as episodes terminated by appropriate ICD therapy (ATP or shock), and a LVEF greater than 35%. Both ischemic and non‐ischemic cardiomyopathies were included. NICM was defined as structural myocardial disease with ventricular arrhythmogenic substrate in the absence of ischemic cardiomyopathy as the primary underlying diagnosis [2]. Patients with congenital heart disease, channelopathies, or primary electrical disease were excluded. Baseline demographic, clinical, imaging, and procedural data were collected from institutional electronic health records at the time of the ablation. For the purpose of this study, the analyzed procedure was the first VT ablation performed at our institution during the study period. Previous VT ablations performed at external centers were recorded as a baseline characteristic. Sustained VT was defined according to the 2022 ESC Guidelines for the management of ventricular arrhythmias and the prevention of SCD [2]. Electrical storm was defined as ≥ 3 VT episodes within 24 h at presentation [2]. The study was conducted in accordance with the principles of the Declaration of Helsinki and was approved by the local ethics committee. Furthermore, this manuscript adheres to the anatomic nomenclature recommended by Anderson, Sternick, et al. for describing the location of substrates associated with abnormal cardiac rhythm [8].

## 3 Electrophysiological Study and Ablation Strategy

Before the procedure, all patients provided written informed consent. The procedures were performed either under conscious sedation with propofol and fentanyl or under general anesthesia, depending on comorbidities and heart failure status. Intravenous heparin was administered to maintain an activated clotting time ≥ 300 s during left ventricular (LV) mapping. Vascular access was obtained via the femoral veins: two 7 F sheaths in the left femoral vein and an 8.5 F steerable sheath (Carto Vizigo, J&J MedTec, Irvine, USA, or Agilis, Abbott, Chicago, USA) was placed in the right femoral vein. A decapolar catheter (Inquiry, Abbott, Chicago, USA) was advanced into the distal coronary sinus, and a quadripolar catheter (Supreme, Abbott, Chicago, USA) was positioned at the right ventricular apex.

Right‐ventricular mapping was performed via the right femoral vein. LV mapping was carried out using a retrograde aortic and/or transseptal approach, with transseptal puncture performed in the anterior‐inferior fossa ovalis under fluoroscopic guidance. An epicardial approach was considered in the presence of ECG, imaging, or unipolar voltage mapping findings suggestive of epicardial or intramural substrate involvement, particularly when endocardial ablation alone was considered unlikely to provide adequate substrate modification. Pericardial access followed the Sosa et al. technique [9], using a subxiphoid puncture and a long steerable sheath (Agilis Epi, Abbott, St Paul, MN, USA).

Electroanatomical mapping (Carto 3, Johnson & Johnson MedTech, Irvine, CA, USA; or EnSite Precision/X, Abbott, Chicago, IL, USA) was performed using dedicated high‐density mapping catheters (OPTRELL Mapping Catheter, OCTARAY Mapping Catheter, Johnson & Johnson MedTech; and Advisor HD Grid Mapping Catheter, Abbott) to assess voltage and chamber anatomy. Low‐voltage areas (< 1.5 mV) were defined as potential arrhythmogenic substrate. Radiofrequency ablation was then performed using open‐irrigated catheters with normal saline (ThermoCool SMARTTOUCH SF/QDOT, Johnson & Johnson MedTech; or FlexAbility, TactiCath, TactiFlex, Abbott).

Programmed ventricular stimulation (drive train S1: 500, 430, 370, and 330 ms, with the addition of up to four extrastimuli) was used to induce the clinical VT [10]. In most patients, VT was present spontaneously or inducible with mechanical stimulation or up to two extrastimuli, and more aggressive stimulation protocols were rarely required. Following substrate mapping and VT induction, activation mapping was performed whenever feasible using multipolar mapping catheters. In hemodynamically tolerated VTs, ablation was aimed at VT termination whenever possible, followed by substrate modification. Pace mapping was used only in selected cases of hemodynamically intolerable VT. In patients without inducible VT, substrate‐based ablation was performed. In cases with an unclear substrate, advanced mapping techniques, including DEEP mapping, were used at the discretion of the operator. Ablation was guided by activation mapping, pace mapping, and substrate modification, targeting the elimination of all identified pathological electrograms. Radiofrequency energy was delivered at power settings of up to 50 W for approximately 60 s per lesion. Advanced ablation techniques such as bipolar ablation, alcohol ablation, or half‐normal saline irrigation were not used.

Following ablation, programmed ventricular stimulation was repeated to assess procedural success. Re‐induction testing was initiated using a stimulation protocol slightly less aggressive than the sequence required for VT induction before ablation and was subsequently escalated stepwise up to 300S4, taking into account hemodynamic tolerance and ventricular refractoriness. The primary procedural endpoint was the elimination of clinical VT. Secondary procedural endpoints included ablation of all inducible non‐clinical VTs and comprehensive substrate modification.

## 4 Follow‐Up

Follow‐up was defined as the period from the first VT ablation procedure in our institution to the last documented clinical evaluation. It included a review of hospital and outpatient records, as well as communication with referring cardiologists and primary care physicians. Patients reporting symptoms suggestive of VT recurrence were evaluated at additional visits. Routine ICD interrogations were performed in all patients with implanted devices to detect asymptomatic arrhythmias. VT recurrence was defined as a documented ventricular tachycardia episode lasting longer than 30 s or resulting in appropriate ICD therapy, where applicable. Antiarrhythmic drug therapy was generally continued after ablation according to institutional practice and was subsequently discontinued at the discretion of the treating physician based on the clinical course and arrhythmia status.

## 5 Statistical Analysis

Continuous variables were reported as mean ± standard deviation, and categorical variables as frequencies or percentages. Continuous variables were compared using the Student's *t*‐test, and categorical variables using the *χ* ^2^ test. Univariate and multivariate Cox regression analyses were performed to identify predictors of VT recurrence. Patients were censored at the time of death, heart transplantation, or last available follow‐up. To avoid overfitting, only variables with *p* ≤ 0.2 in univariate analysis were included in the multivariable model to determine hazard ratios (HRs) and 95% confidence intervals (CIs). A *p*‐value ≤ 0.05 was considered statistically significant. Recurrence rates were calculated and graphically illustrated using Kaplan–Meier curves. All analyses were performed using SPSS version 24.0 (IBM Corp., Chicago, IL, USA).

## 6 Results

### 6.1 Patient and Procedural Characteristics

A total of 219 patients with SHD and LVEF > 35% underwent VT ablation, including 89 (41%) with ICM and 130 (59%) with NICM. Among patients with NICM, the underlying etiology was heterogeneous. NICM with a dilated phenotype or secondary dilated cardiomyopathy was present in 80 patients, arrhythmogenic right ventricular cardiomyopathy in 15, hypertrophic cardiomyopathy in 10, post‐myocarditis cardiomyopathy in 13, laminopathy in 2, and mitral annular disjunction in 1 patient. Scar etiology remained unclear in 9 patients with a non‐dilated phenotype. Compared with NICM, ICM patients were older (67 ± 10 vs. 62 ± 14 years, *p* = 0.002) and more frequently hypertensive (79% vs. 45%, *p* < 0.001), with a slightly lower mean LVEF (42 ± 6% vs. 48 ± 8%, *p* < 0.001). Nearly all patients (98%) carried an ICD at baseline, with single‐chamber devices more frequently used in ICM (55% vs. 39%, *p* = 0.026). Overall, 21 patients (10%) had received an ICD for primary prevention (8 ICM, 13 NICM), most commonly in the context of previously documented lower LVEF values and subsequent improvement in ventricular function over time. Among the few patients without an ICD at the time of the first VT ablation in our institution, device implantation was performed before hospital discharge according to secondary‐prevention recommendations. Detailed baseline characteristics are presented in Table 1.

**Table 1:** Baseline characteristics.

Variable	Total (*n* = 219)	ICM (*n* = 89)	NICM (*n* = 130)	*p*‐value
Age (mean, ±SD, years)	64 ± 13	67 ± 10	62 ± 14	**0.002**
Male gender (%)	191 (87)	79 (89)	112 (86)	0.570
Art. hypertension (%)	129 (59)	70 (79)	59 (45)	**< 0.001**
Diabetes mellitus (%)	30 (14)	14 (16)	16 (12)	0.469
Severe renal dysfunction (%)	21 (10)	11 (12)	10 (8)	0.249
Atrial fibrillation (%)	85 (39)	31 (35)	54 (42)	0.317
LVEF (mean %, ±SD)	45 ± 8	42 ± 6	48 ± 8	**< 0.001**
BMI (mean, ±SD)	27 ± 8	27 ± 8	27 ± 9	0.416
Antiarrhythmic drug (%)	151 (69)	58 (65)	93 (72)	0.317
Previous ablation (%)	75 (34)	28 (32)	47 (36)	0.472
ICD type (%)	214 (98)	86 (97)	130 (100)	**0.026**
1‐chamber (%)	100 (46)	49 (55)	51 (39)	
2‐chamber (%)	61 (28)	22 (25)	39 (30)	
3‐chamber (%)	41 (19)	15 (17)	26 (20)	
s‐ICD (%)	2 (1)	0 (0)	2 (2)	
LBB‐pacing (%)	10 (5)	0 (0)	10 (8)	
Primary ICD indication	21 (10)	8 (9)	13 (10)	0.225
Secondary ICD indication	196 (90)	79 (89)	127 (90)	
ATP only (%)	54 (25)	27 (30)	27 (21)	0.107
ATP + shock (%)	167 (76)	72 (81)	95 (73)	0.182
Electrical storm (%)	51 (23)	18 (20)	33 (20)	0.375

*Note:* Bold value are statistical significance *p* < 0.05.

Procedurally, almost all ICM cases were treated with an endocardial‐only approach (99%), while combined endo‐epicardial ablation was required in 28% of NICM patients (*p* < 0.001). Activation mapping was performed in 195 patients (89%), whereas pace mapping was required only in selected cases of hemodynamically intolerable VT. In 24 patients (11%), VT was not inducible during the procedure and substrate‐based ablation was performed. The median radiofrequency ablation time was 47 min. Acute non‐inducibility of any VT (clinical or non‐clinical) was achieved more frequently in ICM (93%) than in NICM (67%, *p* < 0.001). The clinical VT remained inducible in one patient at the end of the procedure, whereas in all other cases, either non‐clinical VTs were inducible or stimulation testing was omitted due to extended procedural duration. Procedural characteristics are summarized in Table 2.

**Table 2:** Procedural and ablation parameters.

Variable	Total (*n* = 219)	ICM (*n* = 9)	NICM (*n* = 130)	*p*‐value
Endocardial ablation (%)	182 (83)	88 (99)	94 (72)	**< 0.001**
Endo + epicardial ablation (%)	37 (17)	1 (1)	36 (28)	
Non‐inducibility at end (%)	170 (78)	83 (93)	87 (67)	**< 0.001**
VT inducible or no testing (%)	49 (22)	6 (7)	43 (33)	
Complications (%)	14 (6)	4 (5)	10 (8)	0.342
Antiarrhythmic at 6 months FU (%)	92 (42)	38 (43)	54 (42)	0.865
VT recurrence (%)	77 (35)	22 (25)	55 (42)	**0.007**
Re‐ablation (%)	32 (15)	8 (9)	24 (19)	0.051

*Note:* Bold value are statistical significance *p* < 0.05.

Periprocedural complications occurred in 6% of patients. Cardiac tamponade was observed in six cases, treated with pericardial drainage in three and surgical revision in the other half. One patient with splenic injury during subxiphoid access required splenectomy. Three patients developed reactive pericardial effusions treated conservatively with corticosteroids. One patient experienced pneumonia in the early post‐procedural period and recovered with antibiotic therapy. Three groin complications were treated conservatively without the need for surgical intervention.

## 7 Postinterventional Outcomes

Over a mean follow‐up period of 1.4 years, VT recurrence was observed in 77 patients (35% of the total cohort). Recurrence occurred significantly more often in the NICM group than in the ICM group (42% vs. 25%, *p* = 0.007). Repeat ablation was performed in 32 patients (15% overall), again more frequently in NICM than in ICM (19% vs. 9%, *p* = 0.051). Before ablation, 151 patients (69%) received antiarrhythmic drug therapy, predominantly amiodarone (*n* = 121), followed by sotalol (*n* = 27), whereas class IC agents were used only rarely (flecainide *n* = 2; propafenone *n* = 1). At 6‐month follow‐up, antiarrhythmic drugs had been discontinued in 127 patients (58%), while 92 patients (42%) remained on antiarrhythmic therapy. Long‐term antiarrhythmic treatment was more common in NICM than in ICM (40 vs. 12 patients).

During follow‐up, two patients died from complications related to cardiac tamponade, one after epicardial ablation and one following right ventricular perforation by an ICD lead. One patient with progressive end‐stage heart failure died from ventricular fibrillation after ICD deactivation. Four patients ultimately required heart transplantation, and three underwent implantation of durable mechanical circulatory support (two LVADs and one RVAD).

## 8 Recurrence Patterns

**Figure 1:**
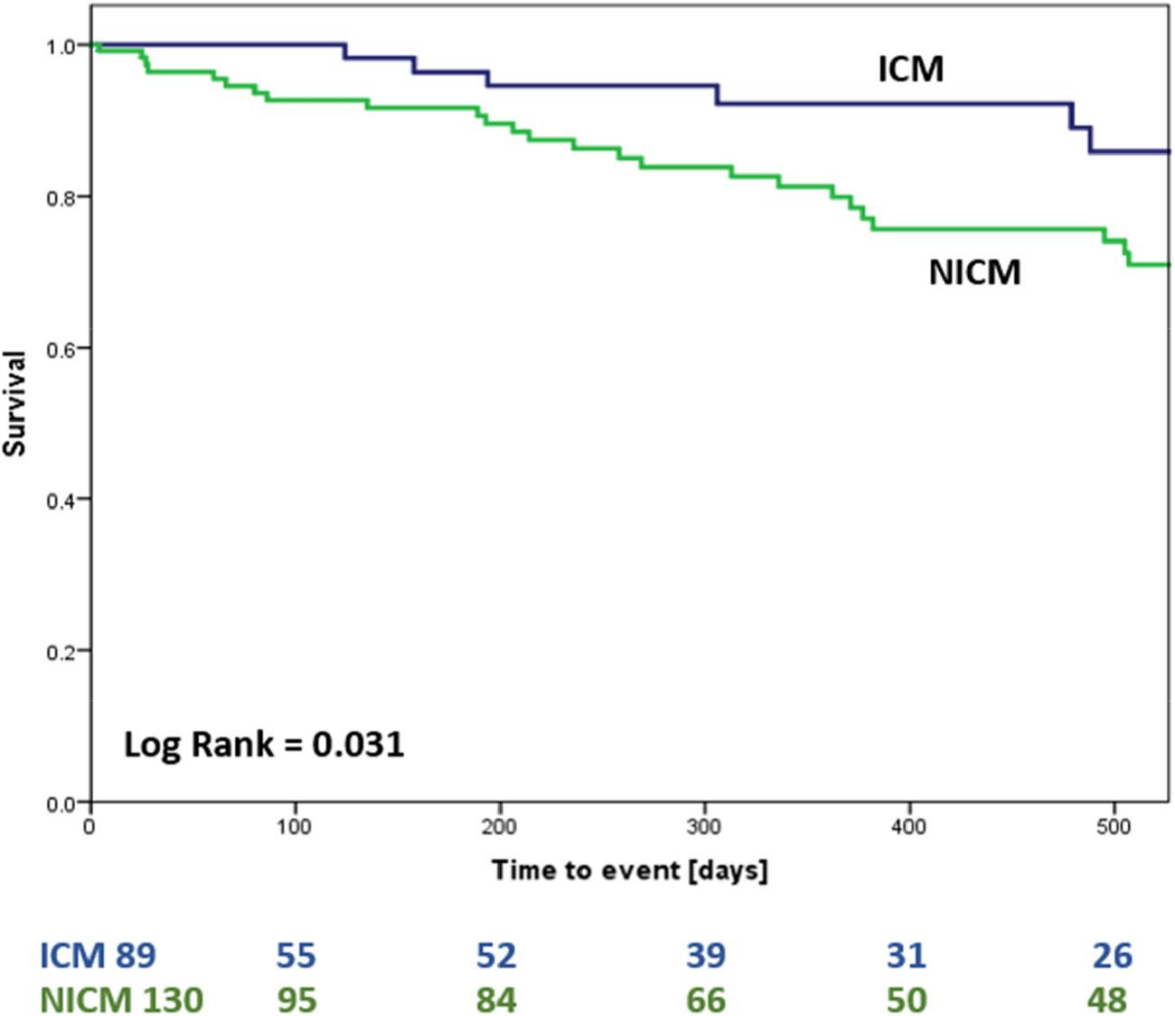
Kaplan–Meier curves showing VT recurrence after ablation in ICM (blue) and NICM (green). NICM patients exhibited a higher recurrence rate, with a significant difference between groups (Log‐rank *p* = 0.031).

In univariate analysis, cardiomyopathy type, BMI, and the use of an epicardial approach were significantly associated with VT recurrence. In the multivariate Cox regression model, only the type of cardiomyopathy was an independent predictor of VT recurrence (HR 2.312, 95% CI 1.3–4.0; *p* = 0.007; Figure 1, Table S1). Acute non‐inducibility at the end of the procedure was associated with a lower risk of VT recurrence (HR 0.54, 95% CI 0.30–0.98; *p* = 0.034); however, this was not reflected by a significant separation of Kaplan–Meier curves (Supporting Information S1: Figure S1).

Among the 77 patients with recurrent VT during follow‐up, the majority of episodes were monomorphic and successfully terminated by anti‐tachycardia pacing (ATP). Forty‐two patients received at least one ICD shock. Malignant VTs with a cycle length < 300 ms were documented in 14 patients (8 NICM, 6 ICM). Ventricular fibrillation or polymorphic VT (CL < 250 ms) constituted the presenting arrhythmia in three patients. In the remaining shock‐treated episodes, ATPs were either ineffective or resulted in acceleration of the initial tachycardia.

**Figure 2:**
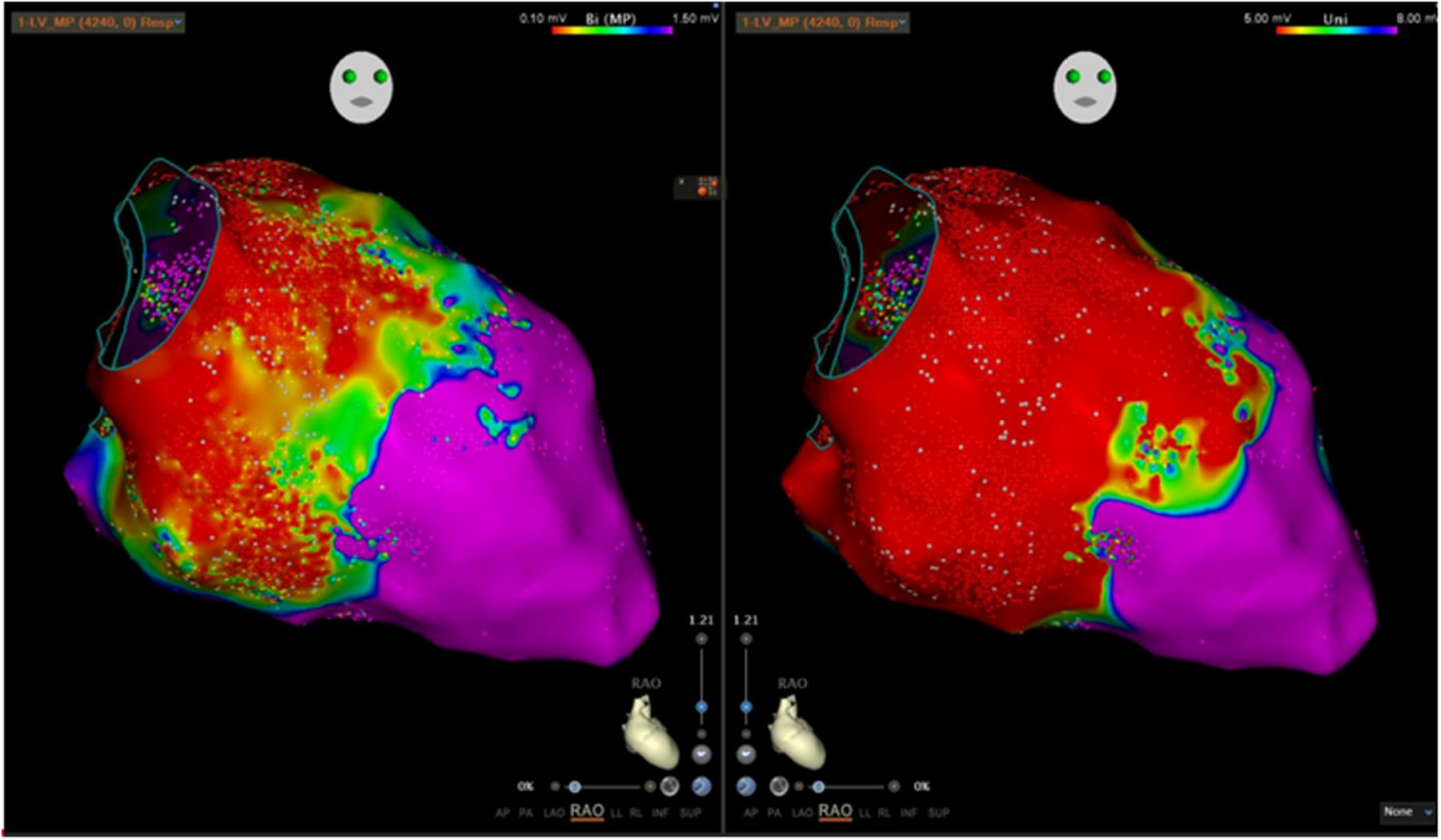
Voltage mapping in a 54‐year‐old male with laminopathy and LVEF 41% referred for VT ablation. Shown is the LV voltage map in right oblique view acquired using the CARTO 3 system. The left panel displays the bipolar voltage map (dense scar < 0.1 mV, low‐voltage area < 1.5 mV), and the right panel the unipolar voltage map (abnormal voltage < 8.3 mV), illustrating the intramural and/or epicardial extension of the scar.

**Figure 3:**
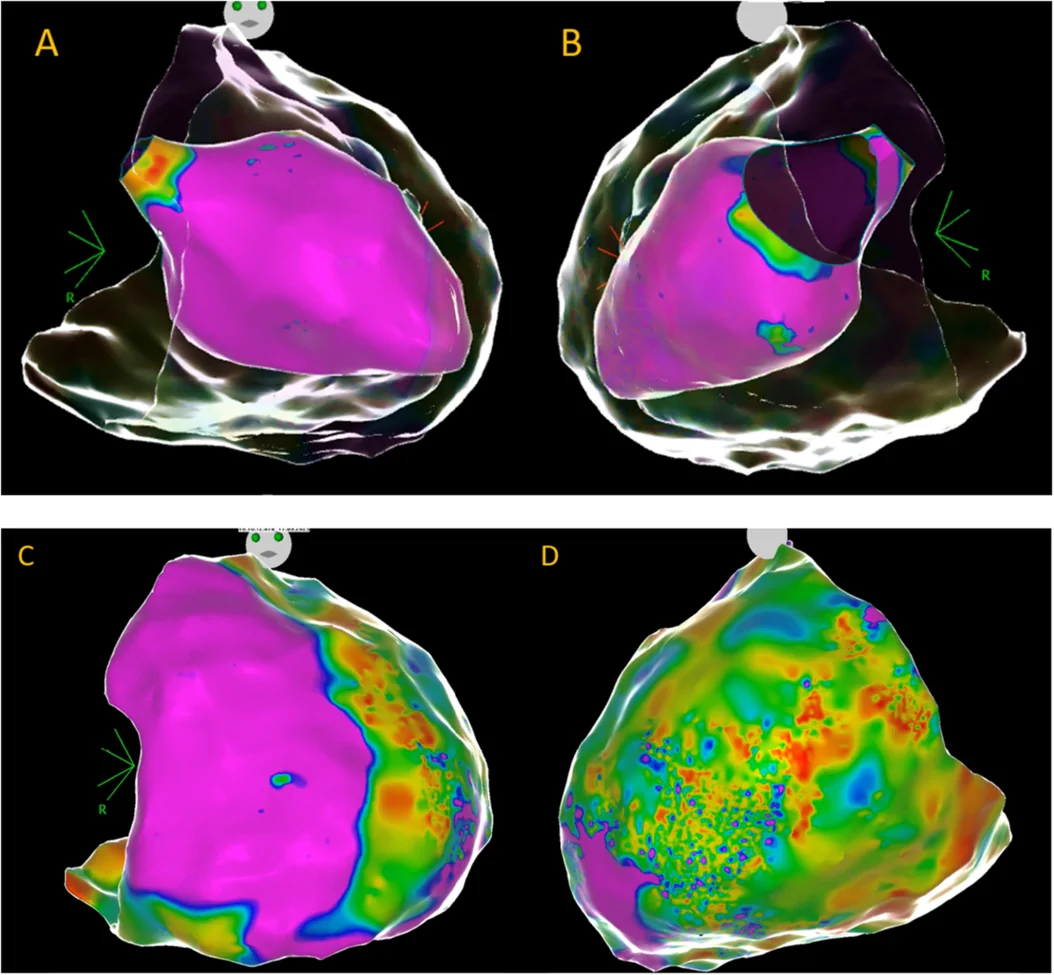
Voltage mapping in a 41‐year‐old male with familial NICM and LVEF 42%. Panels A and B show the endocardial bipolar voltage maps (dense scar < 0.1 mV; low‐voltage area < 1.5 mV) obtained with the CARTO 3 system in right oblique and posteroanterior views, demonstrating no detectable endocardial scar. Panels C and D display the corresponding epicardial bipolar voltage maps (dense scar < 0.5 mV; low‐voltage area < 1.5 mV), revealing a large epicardial scar associated with multiple VT morphologies.

To further characterize substrate differences associated with recurrence, electroanatomical maps were reviewed qualitatively. A broad spectrum of scar distributions was observed across the cohort. In several patients with VT recurrence, scar involvement extended beyond a single LV segment and included basal, lateral or peri‐annular regions, as well as intramural or epicardial components (Figures 2 and 3). In contrast, particularly among ICM patients, scar patterns more frequently appeared localized and confined to discrete endocardial LV regions.

## 9 Discussion

All patients in the present cohort presented with sustained VT and therefore represented a secondary‐prevention population. This study highlights the following major findings in this specific population:

• Despite similar LVEF, VT recurrence after catheter ablation differed substantially according to cardiomyopathy etiology, with significantly higher recurrence rates in NICM compared with ICM.

• VT ablation demonstrated an acceptable safety profile and moderate long‐term arrhythmia control in this secondary‐prevention population with LVEF > 35%.

• In ICM patients with preserved LVEF presenting with hemodynamically tolerated VT, outcomes after catheter ablation were more favorable, underscoring the need for individualized arrhythmic risk assessment beyond LVEF alone.

In this contemporary cohort, VT ablation demonstrated an acceptable safety profile and moderate long‐term arrhythmia control, with clear differences between ICM and NICM. NICM patients experienced higher recurrence rates and required more repeat procedures than those with ICM, consistent with prior reports [2, 3, 11, 12]. These findings underscore that LVEF alone does not adequately capture post‐ablation arrhythmic risk in this secondary prevention population and highlight the central role of arrhythmic substrate characteristics and cardiomyopathy etiology in determining long‐term outcomes. Acute non‐inducibility was associated with lower recurrence risk in multivariable analysis; however, this effect was modest and not reflected by a significant difference in Kaplan–Meier survival estimates.

The less favorable outcomes observed in NICM appear to be closely related to the nature of the underlying arrhythmic substrate. In our cohort, NICM patients more frequently exhibited diffuse, multisegment basal and/or lateral scarring, peri‐mitral involvement, LV summit extension, and epicardial or intramural fibrosis—features that limit endocardial accessibility and provide multiple potential conduction pathways [13, 14], Figure 3]. These observations are consistent with prior data demonstrating that NICM substrates often extend into mid‐myocardial and epicardial layers, making durable substrate modification more challenging [14]. In addition, VT recurrence in NICM has been linked to ongoing myocardial remodeling, suggesting that disease progression may contribute to the need for repeat ablation procedures in this population [11, 12].

Conversely, ICM patients in our cohort more commonly demonstrated localized endocardial scars within predictable coronary territories, most often involving inferior or inferolateral regions. Such substrates are generally more accessible to targeted ablation and may help explain the lower recurrence rates observed in this group [6, 7]. Although the patients in our cohort represent a secondary‐prevention ICD population, the present findings highlight the prognostic importance of successful VT ablation in ICM patients with LVEF > 35%. Our results align with those of Rademaker et al., who reported favorable long‐term outcomes in post‐MI patients with LVEF > 35% undergoing first‐line ablation [7, 15]. Furthermore, prior studies have demonstrated reductions in VT recurrence, ICD therapies, and HF hospitalizations when ablation is performed early [1, 5, 6]. Building on this evidence, a randomized trial is currently evaluating the role of preventive VT ablation in ICM patients [16].

The findings of this study also have broader clinical implications. Current guideline recommendations emphasize comprehensive substrate assessment and individualized arrhythmic risk stratification when planning post‐ablation management and follow‐up in patients with SHD [2, 3]. In NICM—where recurrent VT and HF events occurred more frequently in our cohort—structured follow‐up strategies, including remote device monitoring when applicable, may be particularly valuable; prior studies have demonstrated that daily telemonitoring can reduce mortality and HF hospitalizations [17]. Notably, all but one patient in this cohort who progressed to advanced HF therapies (LVAD implantation or heart transplantation) had NICM, underscoring that a “moderately reduced” LVEF does not necessarily reflect overall disease severity. Accordingly, early optimization of HF therapy, multidisciplinary follow‐up, and timely referral to specialized HF programs remain essential in this population [3, 11].

Future prospective studies are warranted to further refine arrhythmic risk stratification and management strategies in patients with SHD and LVEF > 35%.

## 10 Limitations

This study has several limitations. First, it is a non‐randomized analysis from a single tertiary referral center, which may introduce referral and selection bias. Second, the follow‐up period was relatively short, and late arrhythmia recurrences may therefore be underestimated. Third, as most patients already had an ICD at the time of ablation, the study provides limited insight into ICD management strategies in this setting. Fourth, advanced cardiac imaging was not available in the majority of the cohort, and standardized quantitative assessment of scar burden or scar size across imaging modalities was therefore not possible. Fifth, the NICM subgroup comprised a heterogeneous population with different underlying etiologies. Consequently, the present study was not powered to assess potential differences in arrhythmic outcomes between individual NICM subgroups. Furthermore, mixed cardiomyopathy was not systematically coded as a separate category and could therefore not be analyzed independently. Finally, antiarrhythmic drug therapy was continued in a substantial proportion of patients after ablation, and detailed longitudinal data regarding optimization of guideline‐directed heart failure therapy were not available because of the retrospective study design. Both factors may have influenced long‐term arrhythmia outcomes.

## 11 Conclusion

VT ablation in patients with SHD and LVEF > 35% showed an acceptable safety profile and moderate long‐term arrhythmia control. Recurrence was more frequent in NICM, whereas ICM patients demonstrated higher acute success and more favorable outcomes, underscoring the heterogeneity of arrhythmic risk in this secondary prevention population. P.S. is Member of Advisory Board for Abbott, Boston Scientific, J&J MedTech and Medtronic. All other authors state: None.

## Funding

The authors have nothing to report.

## Disclosure

C.S. received research support and lecture fees from Medtronic, Abbott, Boston Scientific, and J&J MedTec; is a consultant for Medtronic, Boston Scientific, and J&J MedTec.

## Supporting information


**Table S1:** Univariate and multivariate analysis for VT recurrence.
**Figure S1:** Kaplan–Meier analysis of VT recurrence according to acute inducibility status; Ind (−): no VT inducibility, Ind (+): VT Inducibility at procedure's end. No significant difference in recurrence‐free survival was observed between groups (log‐rank *p* = 0.438).

## Data Availability

Data will be made available from the authors on reasonable request.
