# Supplementary material for: Ventricular Tachycardia Ablation in Structural Heart Disease With LVEF > 35%: Clinical Outcomes and Recurrence Patterns
Source: J Cardiovasc Electrophysiol. 2026 Jul 19;37(9):2031–8. doi: 10.1111/jce.70455 (PMC13576576; doi:10.1111/jce.70455)
Supplement: Supplementary file 1 [file JCE-37-2031-s001.docx]

**Online Supplement**

Table S1: Univariate and multivariate analysis for VT recurrence.

| **Variable** | **Total**  **(n**▒=▒**219)** | **No VT recurrence**  **(n**▒=▒**142)** | **VT**  **recurrence**  **(n**▒=▒**77)** | ***p*-value**  **univariate** | ***p*-value**  **multi-variate** |
| --- | --- | --- | --- | --- | --- |
| ICM (%)  NICM (%) | 89 (41)  130 (59) | 67 (47)  75 (53) | 22 (29)  55 (71) | **0.007** | **0.004**  **HR 2.312**  **(CI 1.3-4.0)** |
| Age (mean, ±SD, years) | 64▒±▒13 | 65▒±▒13 | 63▒±▒14 | 0.216 |  |
| Male gender (%) | 191 (87) | 123 (87) | 68 (88) | 0.720 |  |
| Art. hypertension (%) | 129 (59) | 85 (60) | 44 (57) | 0.696 |  |
| Diabetes mellitus (%) | 30 (14) | 20 (14) | 10 (13) | 0.822 |  |
| Severe renal dysfunction (%) | 21 (10) | 14 (10) | 7 (9) | 0.854 |  |
| Atrial fibrillation (%) | 85 (39) | 53 (37) | 32 (42) | 0.539 |  |
| LVEF (mean %, ±SD) | 45▒±▒8 | 45▒±▒8 | 45▒±▒9 | 0.718 |  |
| BMI (mean, ±SD) | 27▒±▒8 | 26▒±▒9 | 29▒±▒8 | **0.047** | 0.379 |
| Secondary ICD Indication (%) | 196 (90) | 125 (88) | 71 (92) | 0.453 |  |
| Antiarrhythmic drug (%) | 151 (69) | 93 (66) | 58 (75) | 0.133 | 0.757 |
| Previous ablation (%) | 75 (34) | 43 (30) | 32 (42) | 0.093 | 0.938 |
| ATP + shock (%) | 167 (77) | 105 (74) | 62 (81) | 0.275 |  |
| Electrical storm (%) | 51 (23) | 32 (23) | 19 (25) | 0.721 |  |
| Endocardial ablation (%)  Endo + epicardial ablation (%) | 182 (83)  37 (17) | 125 (88)  17 (12) | 57 (74)  21 (26) | **0.030** | 0.938 |
| Non inducibility (%)  VT inducible/no test (%) | 170 (78)  49 (22) | 115 (81)  27 (19) | 55 (71)  22 (29) | 0.105 | **0.034**  **HR 0.540**  **CI 0.3-1.0** |
| Complications (%) | 14 (6) | 8 (6) | 6 (8) | 0.533 |  |

**Figure S1.** Kaplan–Meier analysis of VT recurrence according to acute inducibility status; Ind (-): no VT inducibility, Ind (+): VT Inducibility at procedure’s end. No significant difference in recurrence-free survival was observed between groups (log-rank p▒=▒0.438).
